# Simulation and analysis of chest loads on crews during Lunar-Earth re-entry returns

**DOI:** 10.3389/fbioe.2024.1375586

**Published:** 2024-03-18

**Authors:** Jiatao Wang, Zhiyong Peng, Yongjie Yao, Qianxiang Zhou, Pan Guo

**Affiliations:** ^1^ Key Laboratory for Biomechanics and Mechanobiology of the Ministry of Education, School of Biological Science and Medical Engineering, Beihang University, Beijing, China; ^2^ Naval Specialty Medical Center, Shanghai, China; ^3^ School of Mechanics and Safety Engineering, Zhengzhou University, Zhengzhou, China

**Keywords:** lunar exploration spacecraft, re-entry load, crews, the mechanical response of chest, injury analysis

## Abstract

The safety of crews is the primary concern in the manned lunar landing project, particularly during re-entry as the manned spacecraft returns from a direct Lunar-Earth trajectory. This paper analyzed the crew’s chest biomechanical response to assess potential injuries caused by acceleration loads during the re-entry phase. Initially, a sophisticated finite element model of the chest was constructed, whose effectiveness was verified by experiments involving vertebral range of motion, rib lateral rupture, and chest frontal impact. The model was then subjected to the return re-entry loads simulating the Apollo and Chang’e 5 T1 (CE-5T1) test returner to specifically analyze the correlation between the acceleration load and the injury of the crew’s chest tissues and organs. The results indicate that the biomechanical response of crew chest bone tissue under the two return missions is within the threshold value and will not directly cause damage. Compared to the Apollo mission, the CE-5T1 mission’s load poses a higher risk to internal organs. These findings can enhance the crew’s safety and provide reliable assurance for future space exploration.

## 1 Introduction

**FIGURE 1:**
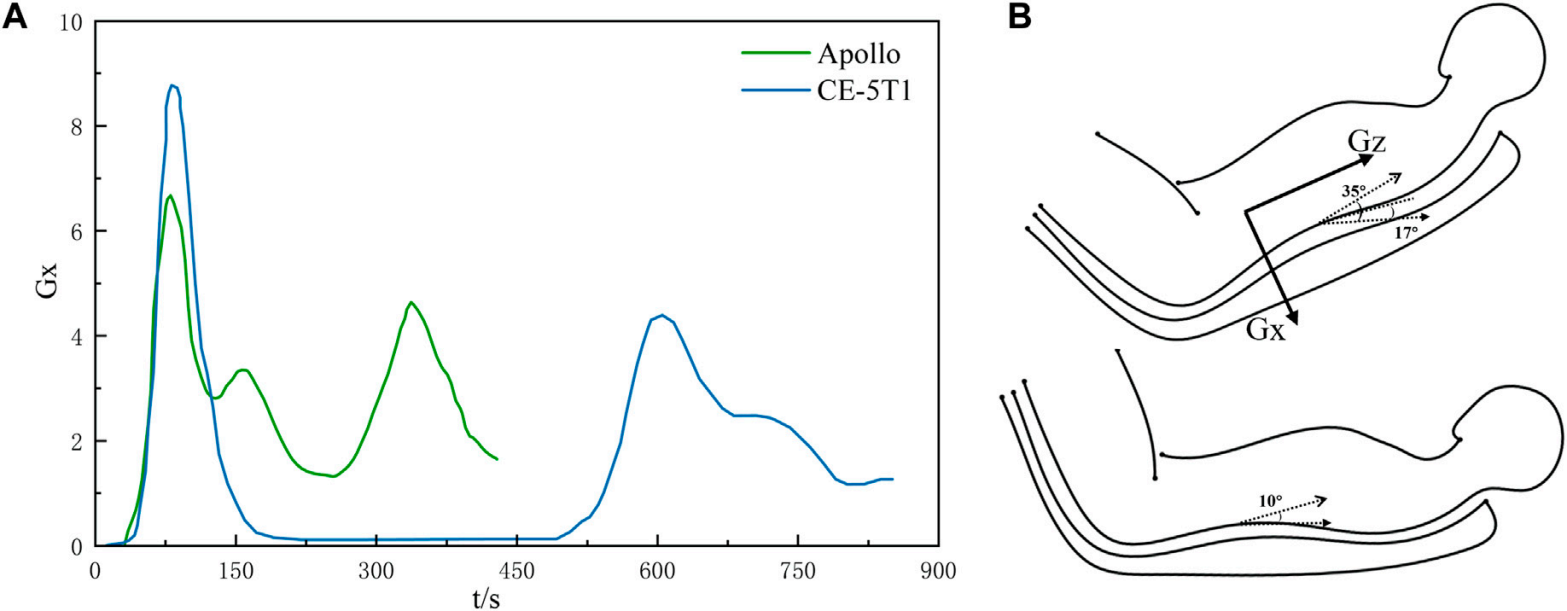
The Lunar-Earth re-entry loads and body postures. **(A)** depicts the acceleration-time curves for Lunar-Earth return loads. The upper portion of **(B)** illustrates the attitude angle range for CE-5T1, where the direction of Gx is chest to back, while the lower portion shows the attitude angle range for Apollo.

Humans have never returned to the lunar surface since the United States achieved manned Moon landings through the Apollo program in the 1970s. With the resurgence of deep space exploration activities in recent years, the National Aeronautics and Space Administration (NASA) has led the formulation of the Artemis program to establish a long-term sustainable human presence on the Moon. China also plans to send crews to the Moon by 2030 to explore the construction of a lunar scientific research and experimental station. The safety and wellbeing of the crews during the spacecraft’s re-entry to the Earth are crucial to manned lunar missions. The acceleration effect on the crew’s tissue injury varies with the different orbital designs of the return module during the re-entry stage. Of the current two main re-entry orbit designs, in Figure 1A, the Sangger ballistic return used by the American Apollo spacecraft reaches double peak accelerations of 6.7 G and 4.5 G, respectively (Graves, 1972); the Orion spacecraft also adopts a similar re-entry strategy, with dual peak values of 6.8 G and 4 G. While the Qian Xuesen-style ballistic return used by the Soviet/Russian Soyuz and the Chinese Shenzhou manned spacecraft reaches double peak accelerations of approximately 9 G and 5 G, respectively, and a prolonged period of near-zero gravity experienced in the Kepler segment between the peaks (Wang, 2012). The acceleration experienced by crews in the return modes mentioned above exceeds the 3–4 G load experienced during low-earth orbit return, which results in a notable increase in musculoskeletal loading, potentially leading to issues such as muscle fatigue, increased skeletal stress, and decreased bone density. Therefore, during the return of China’s manned Moon landing mission, crews faced even higher load levels and a more complex working environment, posing significant challenges to the protection of the wellbeing of the crews.

Body posture is an essential factor directly influencing the human body’s ability to tolerate acceleration. Throughout various stages of spacecraft launch and return, the seat angle of manned spacecraft was frequently selected to modify crews’ body posture in response to varying acceleration loads. As shown in Figure 1B, the Soyuz spacecraft typically employs a recumbent position during launch or atmospheric entry, with the angle between the back and the flat floor normally ranging from 17° to 35° (Liu et al., 2008; Fu et al., 2017). Conversely, the Apollo spacecraft utilizes a lying body position, inclined between 0° and 10° concerning the ground (Graves, 1972). These positions have been identified as effective in enhancing crews’ tolerance to accelerations (Ma et al., 2022).

Although several countries worldwide have been researching the effects of acceleration on the human within manned lunar landing projects, this field is still in its early stages, particularly regarding the precise assessment of potential injuries during missions, which requires further exploration. Previous research has demonstrated that extended exposure to acceleration load beyond a specific threshold can result in various injuries to humans musculoskeletal system, including vertebral fractures, intervertebral disc ruptures, organ contusions, muscle injuries, and ligament tears (Sun, 2005). High + Gx load can also elevate crews’ heart rate, reduce the anteroposterior diameter of the thorax, and significantly diminish chest volume, leading to symptoms such as restrictive dyspnea, hemoptysis, and chest pain (Little et al., 1968). Furthermore, solid organs such as the liver and spleen are at a significantly higher risk of injury under dynamic impact loads than hollow organs like the stomach (Chaudhary et al., 2020). One of the urgent problems in the manned space flight project is to explore the injuries of various tissues and organs of crews caused by re-entry loads, compare the advantages and risks of different Lunar-Earth return schemes, and improve the corresponding injury evaluation standards and protection measures.

Hence, this study constructs a sophisticated finite element (FE) model to examine injuries in chest tissues by calculating the biomechanical response of the crew’s chest during the re-entry load of the Apollo spacecraft and the Chang’e 5 T1 (CE-5T1) test returner, which compares the advantages and risks of the two schemes. The research findings are expected to provide a theoretical basis for designing and improving crew protection devices to reduce chest injuries sustained by crews during re-entry returns.

## 2 Methods

### 2.1 Finite element modeling

Based on the CT data and anatomical structure of the human chest, the finite element model with high biological fidelity was constructed by Mimics 21.0 and Geomagic Studio 2014, including vertebrae, ribs, sternum, and soft tissues such as costal cartilage, intervertebral disc, and ligament.

The CT data was collected from Chinese male taikonaut volunteers who met the criteria of having a height of 171 cm and a weight of 70 kg and did not have any chest lesions, deformities, or other injuries. Data was collected using a 32-slice spiral CT, and the thoracic spine data were explicitly chosen for this study.

Initially, the data was imported into Mimics 21.0 medical software, and three-dimensional point cloud models of various tissues in the chest were generated by adjusting the grayscale values representing tissue density. Subsequently, the models were imported into Geomagic Studio 2014 for smoothing and “grid doctor” inspection and then exported as a geometry model using Nurbs surface. The intervertebral disc boundary was cut out along the bottom surface of the adjacent vertebrae. Then the intervertebral disc was obtained by shelling and Boolean operations, and then the intervertebral disc was divided into the fibrous annulus and the nucleus pulposus by scaling. Due to the complex physiological structure of the human body, there are inherent gaps and connections between tissues, making complete reconstruction based on the internal environment and constraints of the chest difficult. To reconstruct the geometry of the organ models, simplifications were made to the models of the heart, liver, and lungs based on reasonable considerations.

The constructed geometric model encompasses thoracic vertebrae T1-T12, ribs, costal cartilage, sternum, 11 intervertebral discs (annulus fibrosus and nucleus pulposus), skin, and several organs such as the heart, liver, and lungs. To maintain the shape of biological tissues, the vertebrae’s cortical bone was represented as shell units. At the same time, the remaining structures were modeled as high-order tetrahedral units. With mesh element sizes varying from 0.1 mm to 2.5 mm and the Jacobian matrix values indicative of element quality surpassing 0.6 within the range of 0–1, a balance was achieved between result accuracy and computational scale. Ligaments were modeled using spring units, including ligamentum flavum, anterior longitudinal ligament, posterior longitudinal ligament, interspinous ligament, and joint capsular ligament.

Accurate characterization of the material properties of biological tissues was crucial for performing finite element simulations. The material parameters utilized in this model were obtained from experimental data in the literature. Refer to Tables 1, 2 for specific details (Ruan et al., 2003; Cai, 2013; Sis et al., 2016). Specifically, the cortical and cancellous bones of the thoracic vertebrae, ribs, and sternum were composed of elastic-plastic materials, while the skin, costal cartilage, and intervertebral discs consist of linear elastic materials. The heart, liver, and other organs were composed of viscoelastic materials.

**TABLE 1:** Material parameters of chest tissue models (Ruan et al., 2003; Cai, 2013).

Tissue	E/GPa	ν	ρ/(kg/m^3^)	σ_s_/MPa	ε/%	G/GPa	Type
Thoracic cortical bone	13	0.3	2000	227	2	1.15	elastoplasticity
Thoracic cancellous bone	1	0.29	1000	70	3	0.01	elastoplasticity
Sternal cortical bone	12	0.3	2000	120	2	1.15	elastoplasticity
Sternal cancellous bone	0.04	0.3	1000	2.2	3	0.01	elastoplasticity
Rib cortical bone	11.5	0.3	2000	125	2	1.15	elastoplasticity
Rib cancellous bone	0.04	0.45	1000	2.2	3	0.01	elastoplasticity
anulus fibrosus	0.42	0.4	1050	—	—	—	elasticity
nucleus pulposus	0.01	0.49	1020	—	—	—	elasticity
costal cartilage	1.2	0.2	1600	—	—	—	elasticity
Skin	0.035	0.42	1000	—	—	—	elasticity

E = elastic modulus; ν = Poisson’s ratio; σ_s_ = yield limit; ρ = density; ε = plastic failure strain; G = tangent modulus.

**TABLE 2:** Material parameters of major thoracic organs (Ruan et al., 2003; Cai, 2013).

Organ	ρ(kg/m^3^)	Short-time shear modulus/MPa	Long-time shear modulus/MPa	Elastic bulk modulus/MPa	Type
Lung	600	0.067	0.065	2.8	viscoelasticity
Heart	1000	0.02	0.075	0.22	viscoelasticity
liver	1100	0.044	0.44	2.6	viscoelasticity

The human body system consists of diverse organizations and complex structures, and the interconnections between different parts vary. During the model assembly process, fixed constraints were applied between the vertebral bodies and intervertebral discs, fibrous annulus and nucleus pulposus, costal cartilage, ribs, and the sternum. Rotatable joints were established between the ribs and vertebrae, while the skin and bone tissues are connected through shared nodes. Frictionless contact was defined among all surfaces within the thoracic cavity, enabling relative motion and energy transfer even under load conditions.

### 2.2 Model validation

The finite element model underwent validation prior to its utilization in calculations. In this study, the vertebral range of motion, the rib lateral rupture, and the chest frontal impact experiment (Kroell et al., 1974; Charpail et al., 2005; Sis et al., 2016) were employed to validate the accuracy and effectiveness of the chest model.

#### 2.2.1 Validation of vertebral range of motion

Sis et al. (2016) utilized *in vitro* biomechanical experiments to characterize the human thoracic spine’s range of motion (ROM). Thoracic vertebra model was constructed for simulation and calculation based on the thoracic spine ROM experiment. All degrees of freedom beneath the lower surface of the T12 vertebra were constrained to serve as boundary conditions for the model. A moment of force of 5 Nm was applied to the upper surface of the T1 vertebra to simulate flexion-extension, lateral bending, and axial rotation of the thoracic spine.

#### 2.2.2 Validation of rib lateral rupture

The primary direction of acceleration during spacecraft re-entry was chest to back, resulting in observable compression of the chest cavity and deformation of ribs. Charpail et al. (2016) executed experiments on human ribs’ lateral rupture behavior to establish local injury criteria and validate the rib finite element model. Therefore, the Charpail experiments were selected to assess the accuracy of the rib model.

**FIGURE 2:**
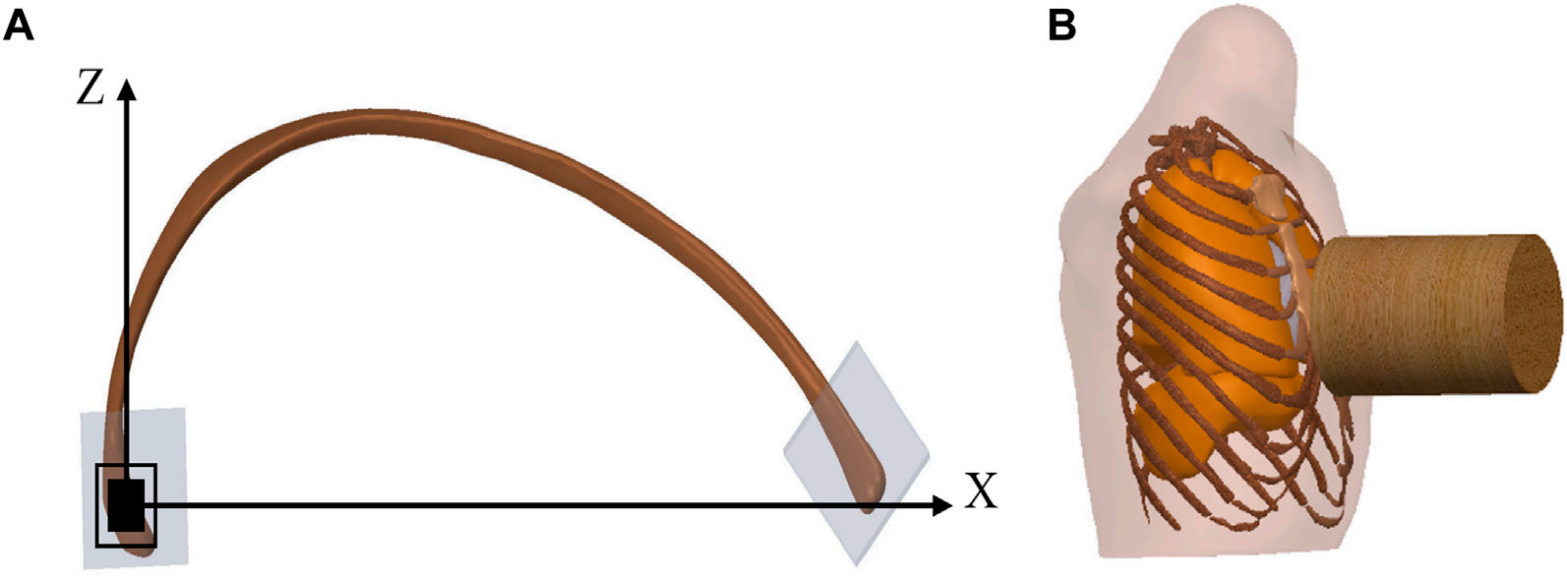
Diagram of verification model. **(A)** illustrates a schematic diagram of structural testing for the fifth rib, where the left end is fixed, and the right end moves towards the negative direction along the *X*-axis. **(B)** illustrates a schematic diagram of the chest frontal impact experiment.

Charpail et al. performed dynamic structural tests and finite element simulation calculations on the left fifth rib. As illustrated in Figure 2A, based on the experimental conditions, the nodal points at both ends of the ribs were embedded in polyester cement. The left end was fixed, while the right end was affixed to the movable track through a pin connection, permitting movement solely along the *x*-axis and rotation around the *x*-axis with negligible friction. A 40 kg pendulum impacted the fixed object at the right rib end, resulting in an initial velocity of 1.8 m/s.

#### 2.2.3 Validation of chest frontal impact

Kroell et al. carried out various experiments to investigate the impact response and tolerance of the human chest (Kroell et al., 1974; Kroell et al., 1971). The variables investigated included the presence of post-mortem corrosion, back restraint, impactor quality, and speed, among other factors. Considering that crews were in a supine posture during the return journey from the Moon to Earth, the validation of chest frontal impact simulation utilized male samples without corrosion treatment and with restrained backs.

Table 3 includedchest frontal impact test data with restraint on the back. The Abbreviated Injury Scale (AIS) was a globally recognized trauma early grading assessment standard based on the severity of injuries and categorizes each type of injury into six levels, ranging from 1 to 6, ascending in severity (including minor, moderate, severe, serious, critical, and extremely critical). The subjects sat upright in front of the impactor, lightly secured to the horizontal support to maintain their trunk posture, and had their backs restrained to prevent upper body rotation upon impact. The wooden impactor used in the experiments was a cylindrical rigid body with a radius of 7.62 cm and a mass of 10.43 kg. It impacted the sternum horizontally between the fourth and fifth ribs at a velocity of 7.2 m/s, as depicted in Figure 2B. The calculation time was set to 60 m.

**TABLE 3:** Kroell chest frontal impact test data (with restraint on the back) (Kroell et al., 1974).

Sample number	Age	Height	Weight	Impact Mass	Impact speed	Maximum force	Deformation	AIS
48FM	69	170	64.4	10.43	7.06	2829	78	0
50FM	66	181	59.9	10.43	7.29	3946	92.2	6
51FM	60	185	82.1	10.43	6.66	2331	78.7	0
52FM	65	175	51.7	10.43	7.20	3051	92.2	4
56FM	65	177	73.9	10.43	6.93	3163	70.4	2
58FM	68	179	68.9	10.43	6.75	2620	76.5	3

### 2.3 Boundary conditions and setup of the computational model

**FIGURE 3:**
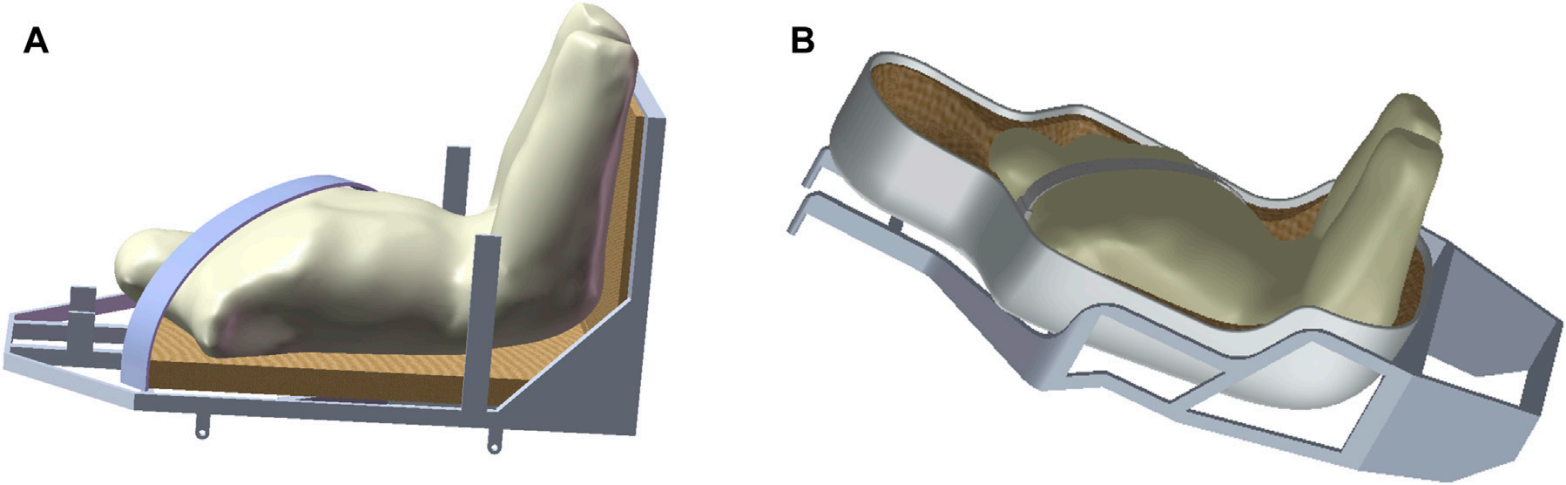
The crew human-seat model, with **(A)** representing the Apollo human-seat model and **(B)** depicting the CE-5T1 human-seat model. The seats have an outer aluminum alloy frame and an inner foam cushion.

The seat angle needed to be adjusted during the re-entry stage of the spacecraft to ensure that crews were in the optimal position to resist the impact of acceleration on their bodies. During the re-entry return process, Apollo crews were in a supine position with a minimal angle between the seat and the floor of the return cabin. The CE-5T1 return scheme was similar to that of the Soyuz spacecraft. To enhance the crews’ ability to withstand acceleration load, the seat angle was set to maintain a specific angle with the floor (Koloteva et al., 2015). As shown in Figure 3, the crew human-seat model utilized an aluminum alloy for the seat, with an elastic modulus of 90 GPa and a yield strength of 0.65 GPa. The framework of the fixed seat served as a constraint for the model. The shaped cushion comprised two materials: foam and an epoxy resin layer. The foam material had an initial elastic modulus of 0.043 MPa and an initial density of 0.078 g/cm³, while the epoxy resin layer material had an elastic modulus of 8 GPa and a density of 1.28 g/cm³. The acceleration curve of the spacecraft during the re-entry return process was depicted in Figure 1. The first peak occurred at a similar time, although with a pronounced difference in peak value, while the second peak appeared at a different time but with similar peak values. The validated model is was imported into Ansys LS-DYNA R12 software for simulating and analyzing the biomechanical response of the crew’s chest.

## 3 Results

### 3.1 Validation results

#### 3.1.1 Validation result of vertebral range of motion

**FIGURE 4:**
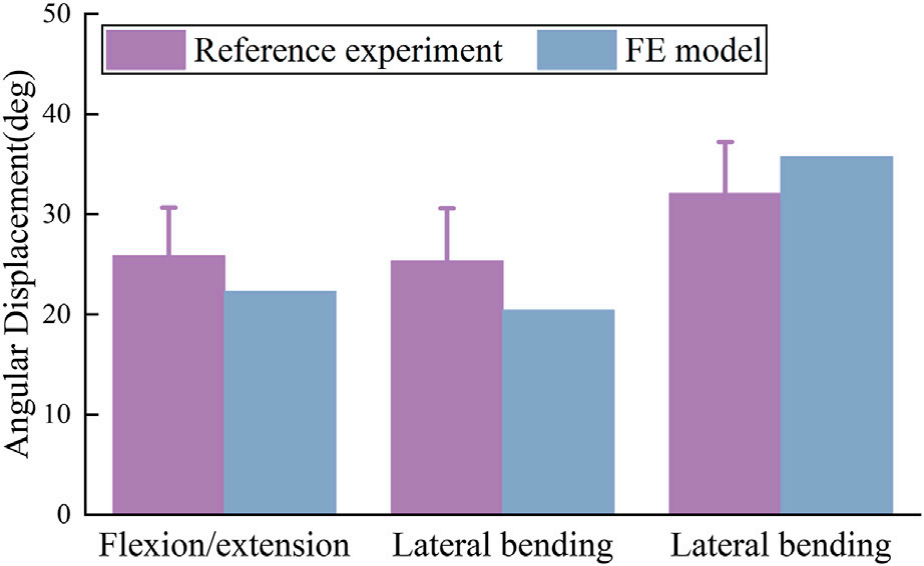
Validation of ROM in thoracic spine FE Model, including flexion-extension, lateral bending, and axial rotation. The simulated activities in the FE model closely match cadaveric experimental data.

The ROM results from simulated analysis are compared with experimental data, as depicted in Figure 4. Compared with *in vitro* experiments, the simulated data closely aligns with the experimental data. Therefore, the model effectively simulates the authentic motion of the thoracic spine under load, and the credibility of the simulated computational results is affirmed.

#### 3.1.2 Validation result of rib lateral rupture

**FIGURE 5:**
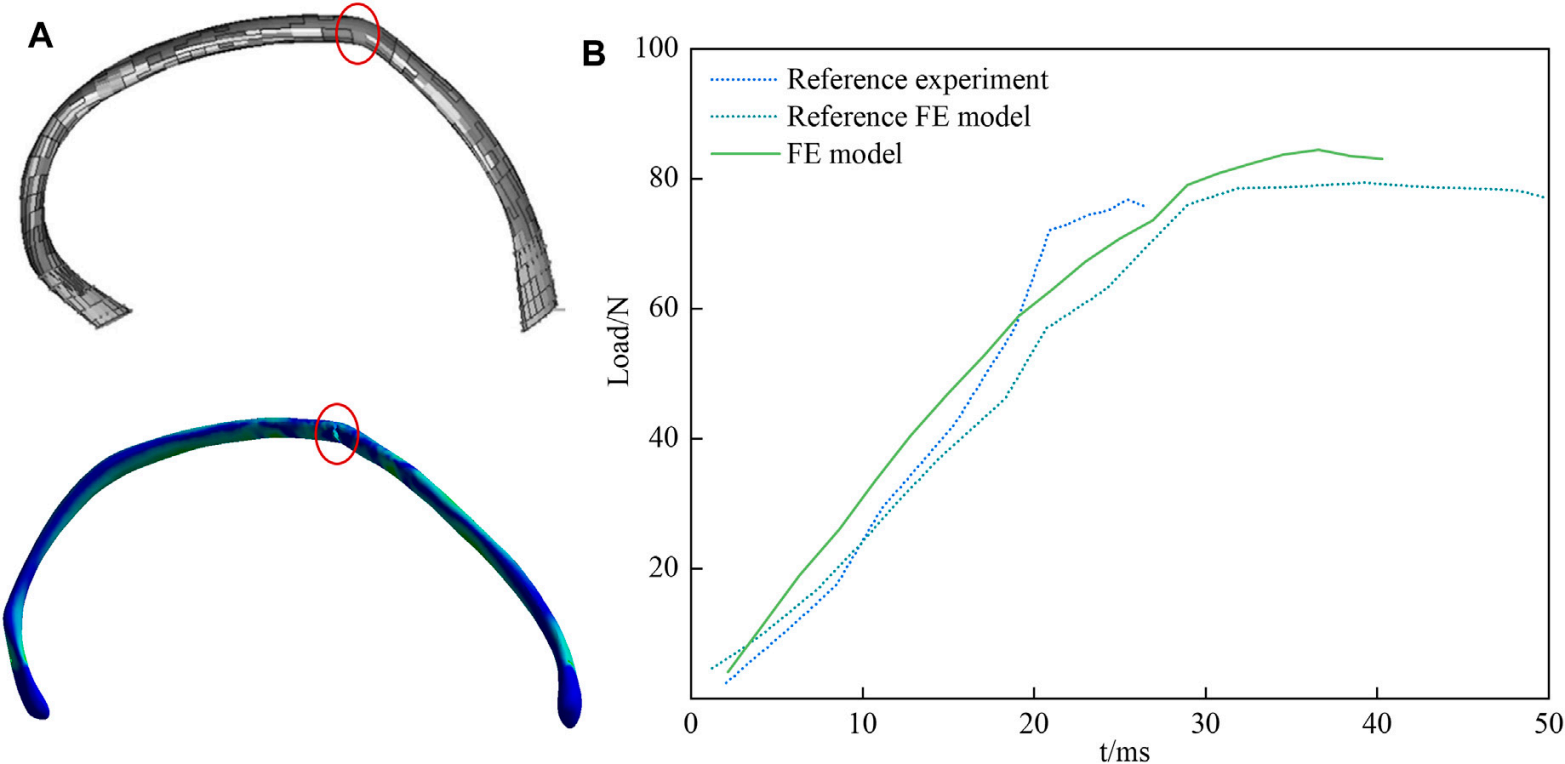
Validation result of rib lateral rupture. In **(A)** the upper part represents the FE model created by Charpail et al., and the lower part is the FE model developed in this study. The rupture location is indicated, and the two models predict the rupture location with good agreement. **(B)** is a comparison of the fracture loads of fifth rib.

The simulated rib fracture time depicted in Figure 5B differs from the target experimental value; nevertheless, the response process generally agrees with the overall trend of the experiment, and the fracture load is relatively similar. The target experiment and simulation yield a value of 78 N, whereas the simulated value in this paper is 84 N, which falls within the acceptable error range. A comparison between the calculated results and the simulation results from the literature in Figure 5A reveals a strong agreement in predicting the location of the fracture between the two models. The above results indicate the effectiveness of the constructed rib model, which can be utilized for the biomechanical analysis of the crew’s chest during re-entry returns.

#### 3.1.3 Validation result of chest frontal impact

**FIGURE 6:**
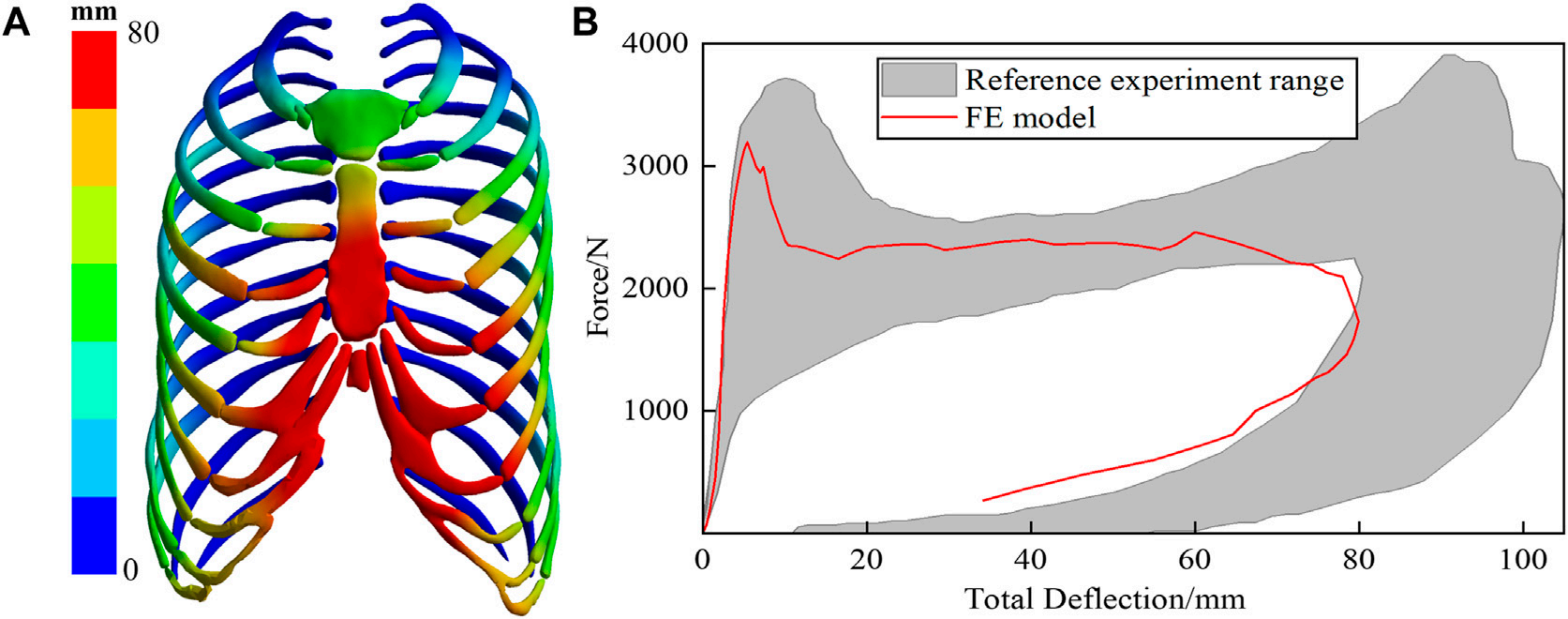
**(A)** depicts the deformation of the ribs. The sternum, impacted during the collision, exhibits significant deformation, particularly in the region associated with the rib cartilage. **(B)** is the chest impact force and total deflection curve.

Figure 6A illustrates the deformation cloud diagram of the chest model. The impacted sternal body exhibits the most remarkable deformation and involves the costal cartilage, reaching a maximum value of 78 mm, which aligns closely with the findings of the comparative experiment. Extract the force-time and deflection-time curves at the central node where the impactor contacts the sternum, Plot the chest contact force-deflection curve, as shown in Figure 6B, and compare it with the experimental range. The simulation curve closely corresponds with the developmental pattern observed in the control experiment. Notably, during the sternum rebound process, a portion of the contact force exhibits relatively high values, which may be attributed to the chest tissue contour, sternum covering, and superficial tissue thickness. The simulation calculation validates the accuracy of the finite element model for the chest under frontal collision, thereby confirming its suitability for analyzing human chest injuries during Lunar-Earth re-entry return.

### 3.2 Calculation result

#### 3.2.1 Calculation result of bone

**FIGURE 7:**
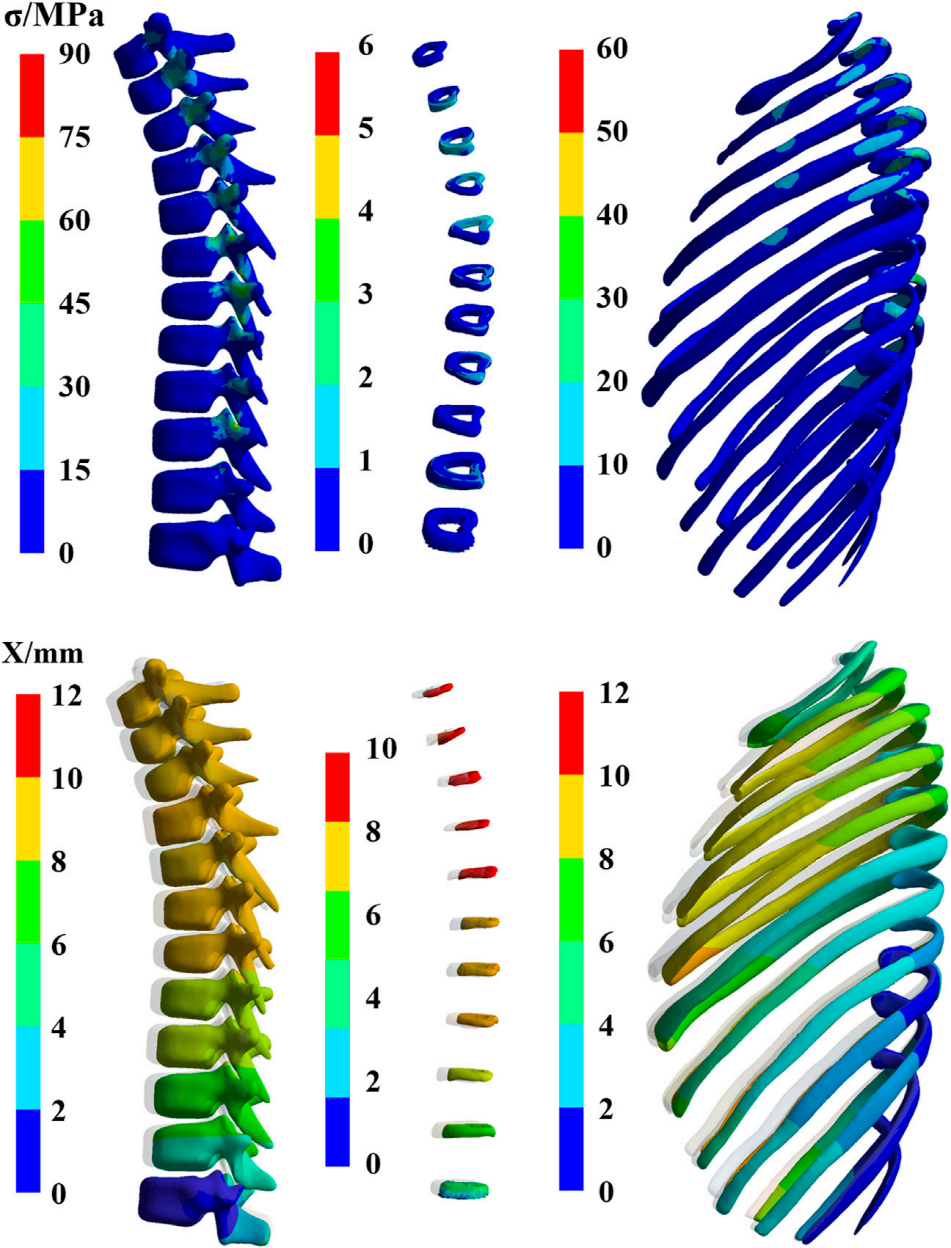
The stress and deformation cloud diagram for bone tissues, including vertebrae, intervertebral discs, and ribs, under the first peak loading condition of the CE-5T1 crews (The shaded part is the original position).

**FIGURE 8:**
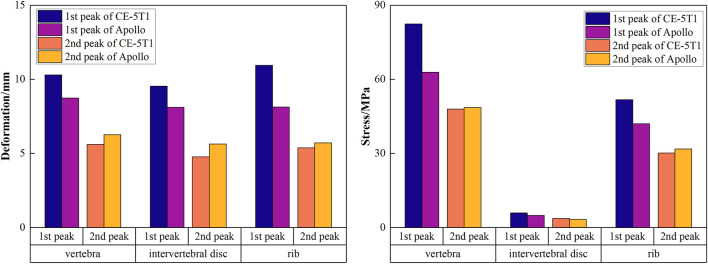
The extreme values of bone tissues for Apollo and CE-5T1 crews under different load peak conditions, including vertebrae, intervertebral discs, and ribs.

Figure 7 presents the stress and deformation cloud diagram for bone tissues, including vertebrae, intervertebral discs, and ribs, under the first peak loading condition of the CE-5T1 crews. The spacecraft seats are equipped with a semi-rigid material called “shaped cushion” to ensure the safety of crews. During re-entry, there is a slight indentation in the overall chest area of the crew, resulting in a vertebral deformation of 10.3 mm for CE-5T and 8.75 mm for Apollo. The regions with the highest stress levels in the thoracic vertebrae are the isthmus and transverse processes of the thoracic vertebrae, making them susceptible to micro-injury of bone tissue. Figure 8 illustrates the extreme values of bone tissues for Apollo and CE-5T1 crews under different load peak conditions. The maximum equivalent stress values for the first and second peaks of CE-5T1 are 82.43 MPa and 47.99 MPa, while for Apollo, they are 62.91 MPa and 48.63 MPa, respectively. The first peak stress of CE-5T1 surpasses that of Apollo, and the stress value is notably higher, while there is no significant difference in stress values for the second peak. Based on the bone strength calculation formula (Carter and Hayes, 1976), the cortical bone strength is 221 MPa. Consequently, the peak stress values of both loads do not exceed the strength limit of the human cortical bone, indicating that they will not directly induce vertebral injury.

**FIGURE 9:**
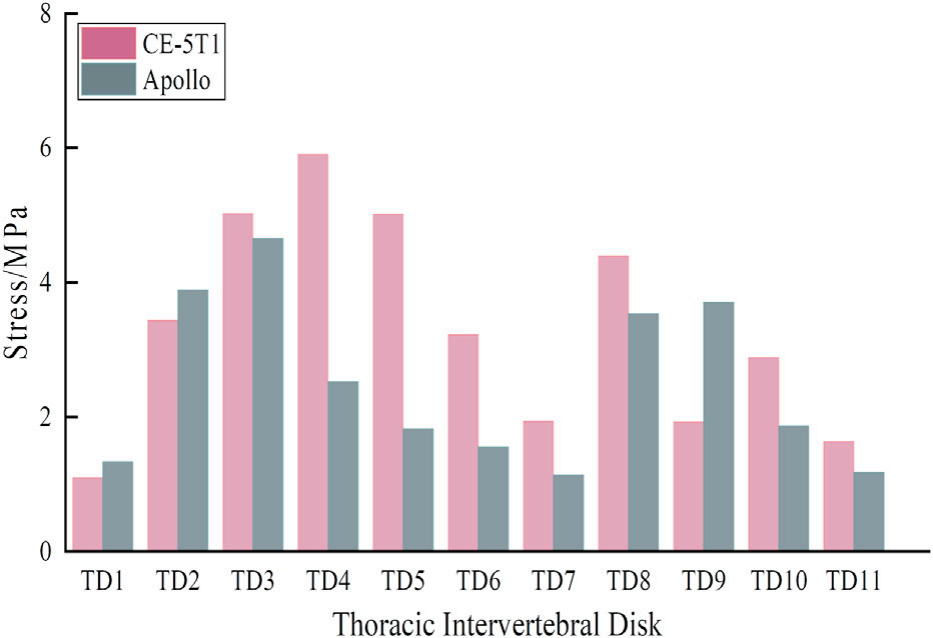
Stress extreme values of each intervertebral disc under different load peak values.

Due to different seating postures, there is a noticeable variation in the maximum stress on intervertebral discs for crews during different return scenarios, with CE-5T1 significantly greater than Apollo. Observing the shape of the thoracic vertebrae reveals varying degrees of inclination of the spinous processes, leading to different terminal positions and causing variations in stress on different intervertebral discs due to contact with the seat. The maximum stress values on intervertebral discs in Figure 9 indicate that during Apollo re-entry returns, TD2, TD3, TD8, and TD9 experience significantly higher stress than other discs. Regarding CE-5T1 re-entry returns, TD2-TD5 and TD8 exhibit higher stress values. The high-strain areas of the intervertebral disc are located on the inner and outer sides of the fibrous annulus. Consequently, the intervertebral disc may degenerate prematurely due to repetitive loading and unloading during daily training and mission operations, leading to impaired spinal function, inevitably impacting crews’ work efficiency and health.

Figure 8 also reveals that the rib’s stress and deformation peak value is higher under the first acceleration peak than the second. The highest stress concentrations in the ribs occur in the rib neck and body of the first, fourth, and fifth sternal ribs. Among them, the range of motion of the sternocostal joint formed by the second rib to the seventh rib and the sternum is not extensive, and the sizeable inertial force makes it subject to a relatively large stress. Based on the stress calculation results, the peak stress values of all ribs are below the bone yield stress of 88 MPa (Li et al., 2010), indicating that severe injuries like fractures and cracks are unlikely to occur. However, the stress concentration areas are susceptible to minor injuries, emphasizing the need for careful attention to protection design.

**FIGURE 10:**
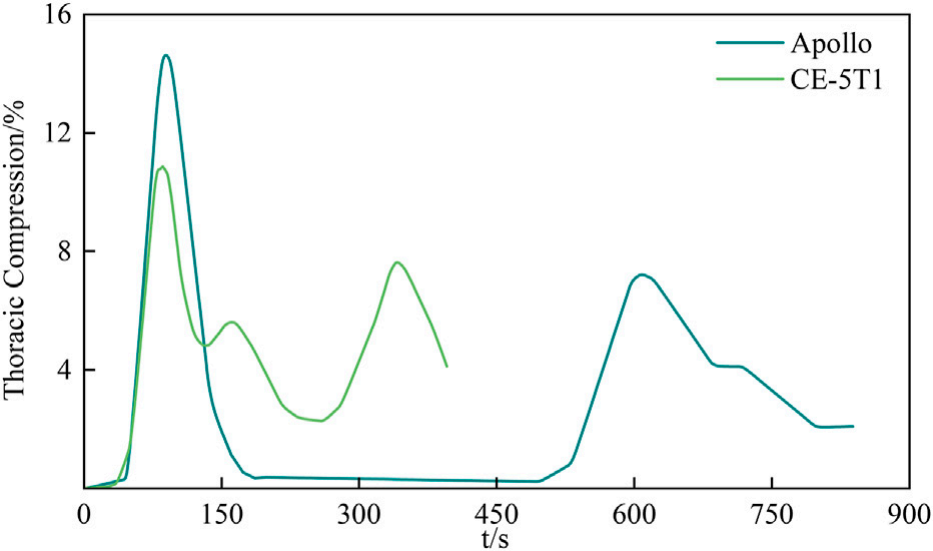
The largest percentage of chest compression at different moments. The maximum compression of the chest is 75mm, with the Apollo crew experiencing a maximum percentage of compression of 10.85%, and the CE-5T1 crew experiencing 14.61%. Both values are significantly lower than 50%, which is indicative of AIS 4 (Severe injuries, potentially life threatening) injuries.

The third and fourth ribs exhibit significantly greater deformation than others. The chest compression injury index defines the occurrence of thoracic fractures, representing the maximum compression deformation of the trunk and ribs, with a threshold of 75 mm. Figure 10 illustrates the results of the maximum chest compression percentage at different moments during the spacecraft re-entry condition. Apollo and CE-5T1 exhibit maximum compression volumes of 10.85% and 14.61%, respectively. Based on the adult compression volume injury guidelines studied by VIANO et al., the risk of chest injury reaching the AIS 4 level is 50%, lower than the allowable chest compression volume threshold (Viano and Lau, 1988).

#### 3.2.2 Calculation result of organs

**FIGURE 11:**
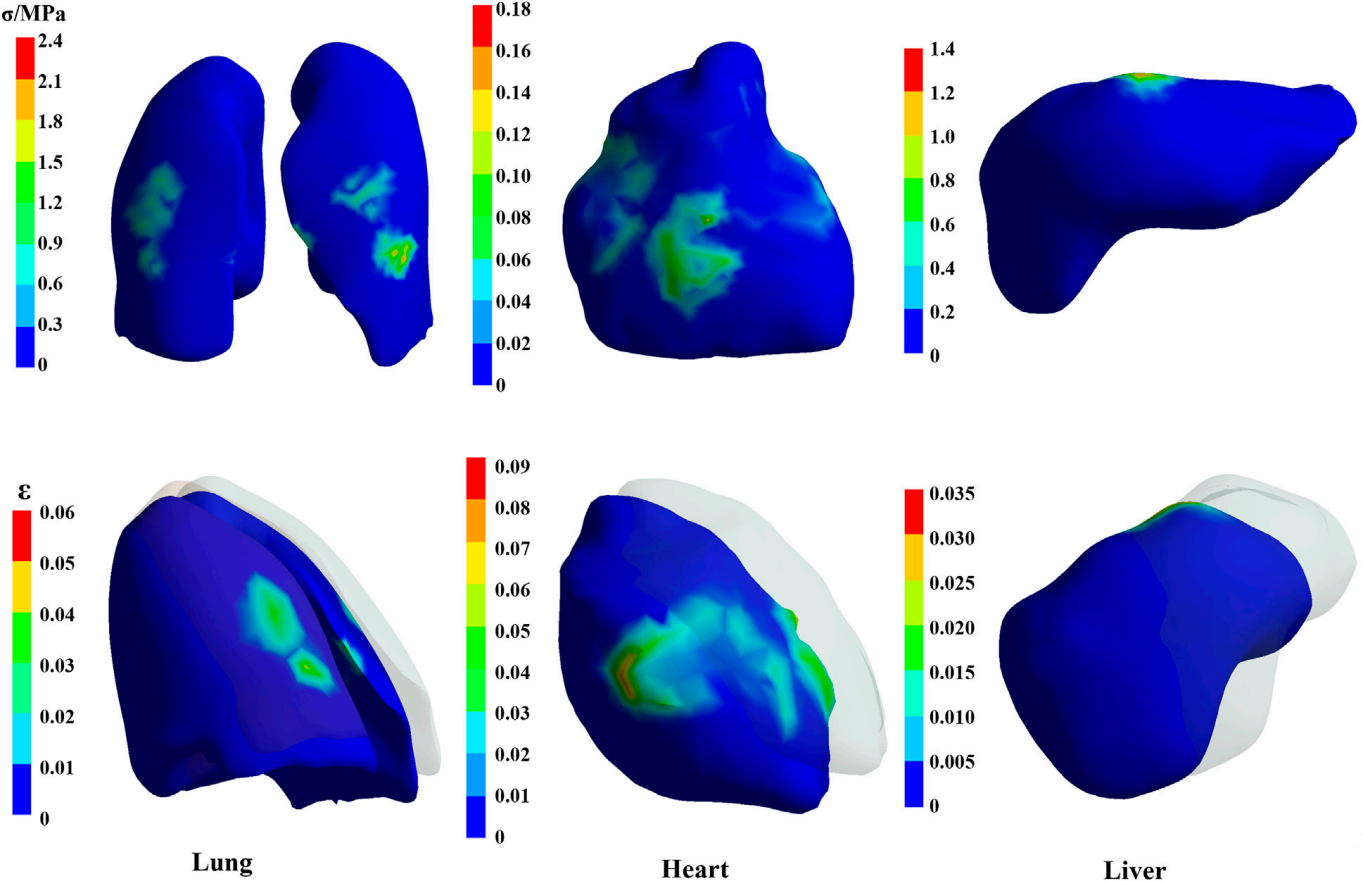
The stress and deformation cloud diagram for organs, including lungs, heart, and liver, under the first peak loading condition of the CE-5T1 crews (The shaded part is the original position).

**FIGURE 12:**
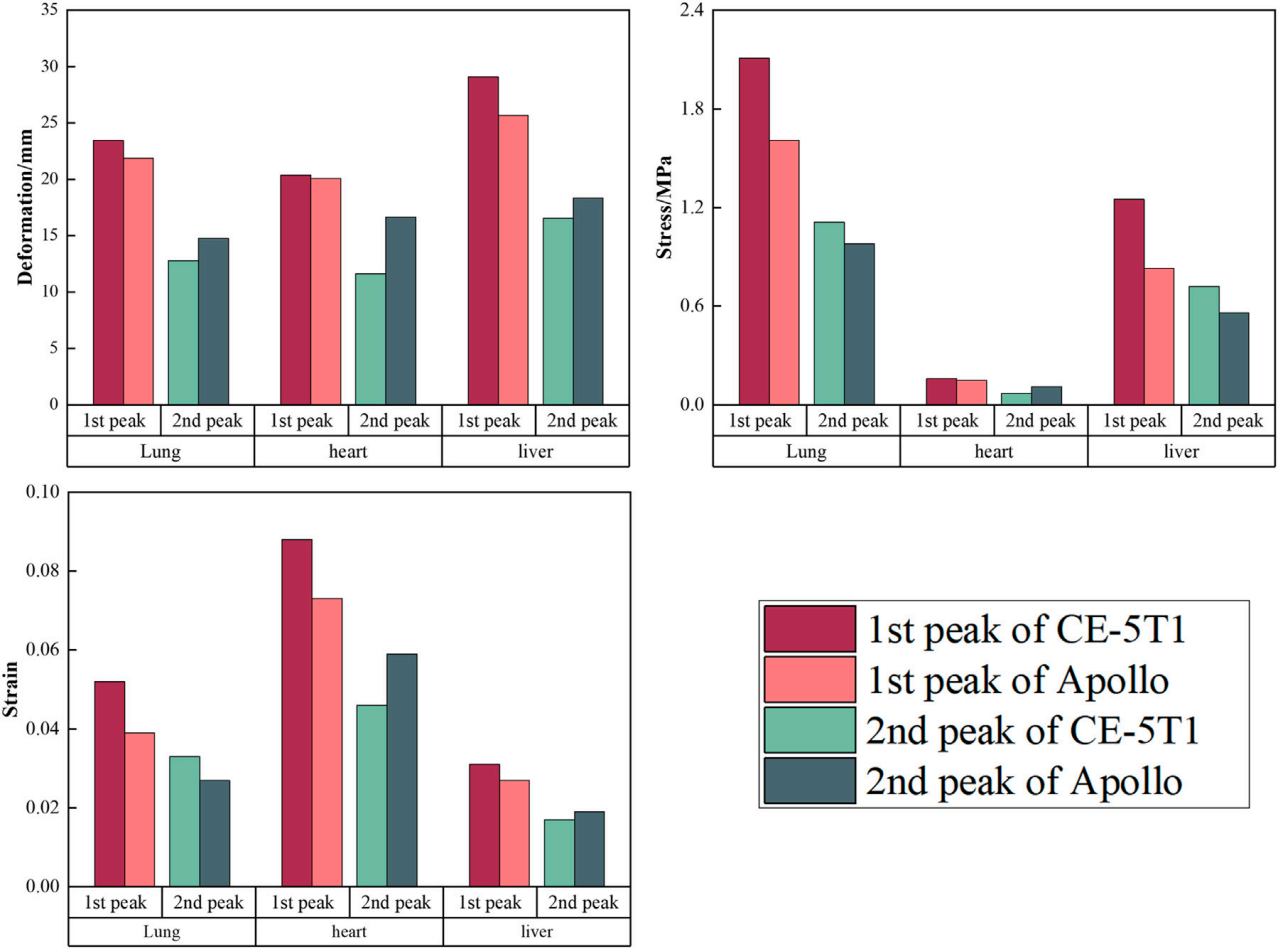
The extreme values of organ tissues for Apollo and CE-5T1 crews under different load peak conditions, including lungs, heart, and liver.

Figure 11 presents the stress and deformation cloud diagram for organs, including lungs, heart, and liver, under the first peak loading condition of the CE-5T1 crews. When subjected to load, the increased inertial forces compress the astronaut’s thoracic cavity and organs, reducing lung capacity, which leads to increased strain on the lower lobe of the left lung and the upper and middle lobes of the right lung, causing increased stress values. Figure 12 illustrates the extreme values of organs tissues for Apollo and CE-5T1 crews under different load peak conditions. The displacements of Apollo at the two peaks are 21.90 and 14.78 mm, with strains of 0.039 and 0.027 and stresses of 1.61 and 0.98 MPa. For CE-5T1, the deformations are 23.45 and 12.77 mm, with strains of 0.052 and 0.033 and stresses of 2.11 and 1.11 MPa. After comparing these values, it was observed that under nearly identical deformation conditions, the stress and strain experienced by the crew’s lungs during the first peak of CE-5T1 were notably higher than those of Apollo. In re-entry missions and other high-load environments, it is essential to prioritize the safety of the lungs, which are susceptible to injury.

The heart is at the center of the chest cavity, slightly leaning to the left, and two lungs surround it. In the model, the heart’s movement primarily relies on the influence of the lungs’ motion and is not influenced by active contraction. Figure 12 illustrates the extreme values of heart deformation under two different load conditions for Apollo and CE-5T1. For Apollo, the deformations are 20.09 and 16.67 mm, with strains of 0.073 and 0.059 and stresses of 0.15 and 0.11 MPa. For CE-5T1, the deformations are 20.36 and 11.61 mm, with strains of 0.088 and 0.046 and stresses of 0.16 and 0.07 MPa. Notably, there is a significant difference in the cardiac response during the second peak between the two load conditions, with CE-5T1 exhibiting significantly lower values than Apollo. This difference arises because CE-5T1 experiences a long duration of near-zero gravity load before the occurrence of the second peak.

The liver is situated in the abdominal cavity, without protection from the thorax, making its structure susceptible to injury from external forces. The liver’s deformation and stress-strain conditions differ between Apollo and CE-5T1 under different load conditions. Specifically, for Apollo, the deformations at the two peaks were 25.69 and 18.36 mm, with strains of 0.027 and 0.019 and stresses of 0.83 and 0.56 MPa. On the other hand, CE-5T1 exhibited deformations of 29.09 and 16.54 mm, with strains of 0.031 and 0.017 and stresses of 1.25 and 0.72 MPa. The difference in liver deformation between the two missions is that CE-5T1 experiences more significant deformation at the first peak. However, the deformation is lower at the second peak than at Apollo.

## 4 Discussion

In this study, a refined biomechanical FE model was constructed based on the anatomical structure of the human chest to better align with the physiology structure. The model’s effectiveness was verified by comparing it with experiments involving intervertebral disc displacement, rib lateral rupture, and frontal impact. After confirming the model’s validity, typical Lunar-Earth re-entry return loads were applied to the model to analyse the risk of crew chest injuries. The study also compared the advantages and risks of different return scenarios, contributing to the refinement of crew safety measures.

The analysis of the simulation results reveals a similar overall trend in the changes of the crew’s thoracic bone tissue under re-entry load conditions, whether during Apollo or CE-5T1 missions. During the first peak condition, CE-5T1 exhibits significantly higher extreme values than Apollo, while at the second peak, CE-5T1 is slightly lower than Apollo due to the longer duration of near-zero gravity close to the Kepler segment. The simulation results indicate that the biomechanical response of crews’ thoracic vertebrae in the space load environment falls within the acceptable tolerance range. With ongoing optimization of flight load design and the implementation of spacesuit protection measures, direct injury to crews’ sternal tissue can be prevented.

Under load conditions, the distinct characteristics of viscera lead to varying patterns of force conduction, resulting in pulling, squeezing, and collisions between viscera and bones, as well as among different viscera, potentially causing visceral damage (Fan and Liu, 2010). Previous investigations on internal organs primarily relied on animal experiments, which frequently failed to replicate the actual flight conditions. NASA has developed a chest injury module utilizing a biomechanical model and a simplified injury scale to calculate the occurrence rate of chest injuries during crew spaceflight on the International Space Station (ISS) (Lewandowski et al., 2012). In this study, using the actual load curve and incorporating the damage model, the results demonstrate that during the first peak, the stress and strain values of CE-5T1 crews’ lungs are significantly higher than those of Apollo due to the enormous first peak acceleration experienced by CE-5T1. Studies have shown that loads below 5g, when crews wear anti-G suits, do not significantly affect pulmonary artery blood flow distribution, while accelerations above 2.5 g can result in dyspnea (Hoppin Jr et al., 1967; Krutz et al., 1990). Symptoms such as autonomous vestibular responses, visual disturbances, and arrhythmia have been predominantly observed among crews of the Soyuz spacecraft during their return to Earth (Koloteva et al., 2015; Kotovskaya et al., 2019; Glebova et al., 2022). To mitigate the impact of the load on the human body, large rigid plates can be added to the chest area of the anti-G suit (Danelson et al., 2011). In contrast, the crews’ heart deformation during the second peak of CE-5T1 was notably lower than that of Apollo. This difference can be attributed to the prolonged exposure to the near-zero gravity Kepler segment experienced by CE-5T1 before the second peak. With the progress of manned spaceflight projects, it is anticipated that crews will experience more severe injuries and irreversible tissue changes. Therefore, spacecraft designers should prioritize the mechanical condition of the internal organs of the human body, particularly during the return phase of a mission. Exposure to the microgravity environment in space diminishes the physical function of crews, compromises their capacity to endure load, and renders them more susceptible to breathing difficulties (Wu et al., 2012; Kotovskaya et al., 2019). H Ma et al. (2022) utilized the HUMOS dummy to develop seat dummy system models representing various human postures. They discovered that the deformation of the diaphragm are critical factors influencing the human body’s tolerance to re-entry load. As discussed in this article, the absence of thoracic protection leads to increased liver deformation during blunt shock load, thereby increasing the likelihood of injury.

A comparative analysis of the physiological effects on internal organs between the two return schemes demonstrates the greater severity induced by CE-5T1. During this period, the combined effects of accelerations in thoracic-dorsal and head-pelvis directions impact the human body. Furthermore, the Kepler segment, which approaches 0g, mitigates the cumulative effect of continuous acceleration on the organs. In terms of safety, taking into account the detrimental effects of space missions on crews’ physical functions, it is advisable to limit the maximum peak of the re-entry load of the CE-5T1 spacecraft returning from lunar orbit to the maximum acceleration peak of the Apollo spacecraft, while maintaining the original Keplerian segments between peaks.

The limitations of this study primarily stem from the simplification and idealization of human anatomical structures and physiological characteristics. During finite element analysis, organs such as the heart and liver were simplified as isotropic ideal entities, neglecting their complex physiological structures. Similarly, material parameters of tissues like intervertebral discs were simplified, overlooking the nonlinear mechanical properties of biological tissues. Such simplifications and idealizations may result in discrepancies between the established models and actual physiological conditions. Consequently, ongoing research will gradually refine these models, enhancing the reliability and applicability of the study findings.

To comprehensively assess the chest’s response during crews’ re-entry missions, developing a multi-scale model of the viscera and conducting in-depth hemodynamic analyses is imperative, enhancing our understanding of the precise impacts and challenges that may arise during re-entry.

## 5 Conclusion

The FE model was constructed to analyse the biomechanical response and tissue injury in crews’ chests during Earth-Moon re-entry load, which can effectively reveal the mechanism of sustained acceleration between tissues. The simulation results demonstrate that the biomechanical response of the crew’s thoracic bone tissue falls within the tolerance threshold under both return schemes, indicating the absence of direct injury. CE-5T1 load presents a higher risk to internal organs compared to Apollo. These findings will enhance crew safety and provide reliable assurance for future space exploration.

## Data Availability

The original contributions presented in the study are included in the article/supplementary material, further inquiries can be directed to the corresponding author.
